# The antioxidant activity of polysaccharides from *Armillaria gallica*

**DOI:** 10.3389/fnut.2024.1277877

**Published:** 2024-02-14

**Authors:** Peiwen Su, Honghao Qiu, Lishan Liang, Luo Weng, Yingjie Liu, Jiajun Liu, Liyan Wu, Fanxin Meng

**Affiliations:** School of Pharmacy and Food Science, Zhuhai College of Science and Technology, Zhuhai, China

**Keywords:** Armillaria gallica, polysaccharides, antioxidant capacity, HepG2, antioxidant

## Abstract

The purpose of this study was to investigate the antioxidant activity of *Armillaria gallica* polysaccharides. It explored whether *Armillaria gallica* polysaccharides (AgP) could prevent HepG_2_ cells from H_2_O_2_-induced oxidative damage. The results demonstrated that HepG_2_ cells were significantly protected by AgP, and efficiently suppressed the production of reactive oxygen species (ROS) in HepG_2_ cells. Additionally, AgP significantly decreased the abnormal leakage of alanine aminotransferase (ALT) and lactate dehydrogenase (LDH) caused by H_2_O_2_, protecting cell membrane integrity. It was discovered that AgP was also found to regulate the activities of antioxidant enzymes, superoxide dismutase (SOD), catalase (CAT), and glutathione peroxidase (GSH-PX), while reducing malondialdehyde (MDA), thus protecting cells from oxidative damage. According to the flow cytometry analysis and measurement of caspase-3, caspase-8, and caspase-9 activities, AgP could modulate apoptosis-related proteins and attenuate ROS-mediated cell apoptosis.

## 1 Introduction

A edible and therapeutic fungus is *Armillaria gallica* Marxm. & Romagn., which is a member of the family Physalacriaceae, order Agaricales, and phylum Basidiomycota. The *Armillaria gallica* plays a crucial role in the growth and reproduction of valuable Chinese medicinal herbs *Gastrodia elata* Bl. and *Polyporus umbellatus* (Pers.) Fr. (1). *Armillaria gallica* is the symbiotic bacteria of *Gastrodia elata* and *Polyporus umbellatus*, which must be infected by Armillaria to grow and reproduce (1). According to studies, *Armillaria gallica* has high nutritional value because contains several substances, including sesquiterpenes, polysaccharides, adenosine, amino acids, and trace elements (2–4). Numerous compounds have been isolated and identified from *Armillaria gallica*, including polysaccharides, erinacine-type sesquiterpenes, phenols, lipids, polyols, organic acids, lipids, purines, and sterols (2–4). Research has demonstrated that *Armillaria gallica* exhibits diverse pharmacological effects, including anti-tumor (2, 4), anti-aging (5), immune-regulating (6, 7), hypoglycemic (8, 9), and neuroprotective effects (10). In the research (Lou et al.) adopted MTT assay with paclitaxel as a positive control and then determined the extent of inhibition of human cancer cells (A549HCT-116, M231 and W256) by compounds 1–6 (4). In another study, polysaccharides activated macrophage-mediated immune responses, generated nitric oxide (NO), reactive oxygen species (ROS), Interleukin-1β (IL-1β), tumor necrosis factor (TNF-α) and Interleukin-6 (IL-6), and enhanced phagocytosis and absorption (Tan et al.) (7). In addition, enriched polysaccharides extracted from *Amillariella mellea* substrates had an oral hypoglycaemic effect at high doses, reducing fasting blood glucose and improving glucose tolerance according to some researchers (Yang et al.) (8).

Polysaccharides are complex high molecular weight compounds are made up of multiple monosaccharide molecules linked together by glycosidic bonds (2, 3). These biopolymers are non-toxic, naturally occurring, and biodegradable and have a variety of useful functional characteristics. Significant biological actions that polysaccharides display include prebiotic, antioxidant, antibacterial, anti-lipid, anti-diabetic, anticoagulant, anti-inflammatory, anti-aging, and anti-tumor properties. These bioactive potentials are essential for boosting immune response (11).

Antioxidant activity plays a vital role in maintaining human health. Free radicals and reactive oxygen species (ROS), which are produced by oxidative processes in the body, can harm functional molecules and cellular structures. Oxidative stress plays a pivotal role in various diseases, including obesity, cirrhosis, diabetes, atherosclerosis, cancer, Alzheimer’s disease (AD), cardiovascular ailments, and reperfusion injury (12, 13). In cells, ROS was generated from a variety of sources and mitochondria played a crucial role in oxidative respiration (12). When present in sufficient amounts, ROS can act as signaling molecules for several cellular functions, such as growth, differentiation, survival, as well as inflammation and immune responses (13). When kept at acceptable levels, ROS produced by cells under normal physiological conditions do not represent a threat to the system since they can be efficiently eliminated by membrane metalloproteinases such as superoxide dismutase (SOD) and heme oxygenase-1 (HO-1) (14). Excessive levels of ROS can cause oxidative damage to crucial biomolecules within cells. The accumulation of ROS triggers oxidative stress (OS), which compromises the integrity of cellular structures and activities by altering DNA, lipids, and proteins. (15) When damaged biomolecules are deposited inside of cells, dangerous compounds like lipofuscin can occur, which can cause metabolic problems, decreased cellular activity, and even cell death. The liver is particularly vulnerable to toxins and oxidative substances since it is in charge of receiving blood from the intestines through the portal vein (14). However, studies have shown that the excessive reactive species such as superoxide radical(O_2_⋅^−^), hydroxyl radical(⋅OH), and H_2_O_2_ significantly leads to the cellular toxicity, resulting in diseases such as steatohepatitis, liver fibrosis, cirrhosis, and hepatocellular carcinoma, among the leading causes of global mortality. (14, 15) Polysaccharides, as natural antioxidants present in various natural products, may effectively alleviate oxidative damage by neutralizing the toxic effects of oxidative free radicals (15).

I This study examined the antioxidant capacity of *Armillaria gallica* polysaccharides (AgP) in HepG_2_ cells exposed to H_2_O_2_-induced oxidative damage. We measured the amount of malondialdehyde (MDA), ROS, apoptotic rate, and intracellular antioxidant enzyme activity. This research showed that AgP acted as a shield against oxidative damage caused by H_2_O_2_, and it may lead to the development of AgP as a naturally occurring antioxidant.

## 2 Materials and methods

### 2.1 Materials and reagents

The Da Hinggan Ling Prefecture of Heilongjiang Province, China, was used as the source for the *Armillaria gallica* strain in this study, and this strain was confirmed to be *Armillaria gallica* by Shanghai Bioscience & Biotechnology Co., Ltd. The HepG_2_ cell line, with the catalog number TCHu 72, was purchased from the Cell Bank of the Typical Culture Preservation Committee of the Chinese Academy of Sciences. Papain, cellulase and pectinase were provided by Shanghai Yuanye Bio-Technology Co., Ltd. (Shanghai, China). High-glucose DMEM culture medium, phosphate-buffered saline (PBS) and penicillin–streptomycin solution were provided by Hyclone (Logan, UT, United States). Trypsin–EDTA cell digestion solution was from Sangon Biotechnology Co., Ltd. (Shanghai, China). Fetal bovine serum (FBS) were bought from Procell Life Science & Technology Co., Ltd. (Wuhan, China). Cell counting Kit-8 (CCK-8) were obtained from the Beyotime Institute of Biotechnology (Nanjing, China). Dimethyl sulfoxide (DMSO), caspase-3 activity assay kit (catalog number: BC3830), caspase-8 activity assay kit (catalog number: BC3880), caspase-9 activity assay kit (catalog number: BC3890), Annexin V-FITC/PI apoptosis detection kit(catalog number: CA1020) and Reactive oxygen species (ROS) assay kit (catalog number: CA1410) were obtained from Beijing Solarbio Science & Technology Co., Ltd. (Beijing, China). Malondialdehyde (MDA; catalog number: A003-4-1) assay kit, alanine aminotransferase (ALT) assay kit (catalog number: C009-22-1), glutathione peroxidase (GSH-PX) assay kit (catalog number: A005-1-2), lactate dehydrogenase (LDH) assay kit (catalog number: A020-2-2), catalase (CAT) assay kit(catalog number: A007-2-1) and superoxide dismutase (SOD) assay kit (catalog number: A001-3-2) were provided by Nanjing Jiancheng Bioengineering Institute(Nanjing, China).

### 2.2 Extraction of polysaccharide from *Armillaria gallica*

The crude polysaccharides were extracted from *Armillaria gallica* under the following conditions: *Armillaria gallica* was dried in an oven at 60°C and then was grinded to a particle size of 80 mesh. Then, it was added with enzymes at a mass ratio of 4% and dissolved in distilled water. The extraction process lasted for 2 h at a temperature of 45°C and at a pH value of 5. The enzyme combination used was papain: cellulase: pectinase in a ratio of 2:2:1. The sample-to-liquid ratio was 1:50 g/mL.

### 2.3 Purification of polysaccharide from *Armillaria gallica*

To remove proteins from the crude polysaccharides, this study employed Savage method with slight modifications. First, the crude polysaccharides extracted from *Armillaria gallica* were dissolved completely in distilled water. The crude polysaccharides was dissolved in distilled water and reached the concentration of 40 mg/mL. The concentration of the crude polysaccharides was tested based on phenol-sulfuric acid method (6). The Sevage working solution was prepared by mixing chloroform and *n*-butanol in a 4:1 (v/v) ratio (6). The Sevage working solution was then added to the polysaccharide solution in a ratio of 4:1 (v/v) and stirred vigorously using a magnetic stirrer for 30 min to ensure full mixing (6). The mixture was then centrifuged at 4,000 rpm for 15 min to separate the protein precipitate layer in the middle and the organic layer at the bottom. The aqueous phase was collected, after the organic and protein precipitate layers were discarded. This process was repeated until no noticeable protein precipitate was observed in the middle layer, and only the water phase was collected. To obtain the *Armillaria gallica* polysaccharides (AgP), the collected water phase was diluted to a concentration of 10 mg/mL using distilled water. The polysaccharide solution was then transferred into a 100 kDa ultrafiltration centrifuge tube and centrifuged at 4,000 rpm for 25 min. The intended *Armillaria gallica* polysaccharides (AgP) were isolated as a result of this procedure, which enabled the separation of the polysaccharides from smaller molecules. The AgP was then lyophilized and stored at the drying oven.

### 2.4 Analysis of monosaccharide composition in *Armillaria gallica* polysaccharides

1. Accurately weigh 10 mg of the lyophilized *Armillaria gallica* polysaccharides into a stoppered vial with a volume of 20 mL. Add 5 mL of 2 mol/L trifluoroacetic acid (TFA) and seal the vial with a nitrogen gas-filled stopper. Place the reaction mixture in a 100°C oven for 2 h to hydrolyze the polysaccharides. After cooling, remove the vial lid.
2. Mix 1 mL of the reaction mixture with 1 mL of methanol and place it in a 70°C water bath while purging with N_2_. Repeat this process twice to remove the TFA.
3. Add 1 mL of 0.3 M NaOH solution to dissolve the residue completely, obtaining the hydrolysate of *Armillaria gallica* polysaccharides for further derivatization analysis.
4. Take 400 μL of a mixed monosaccharide standard solution and the hydrolysate of *Armillaria gallica* polysaccharides, and transfer them into separate stoppered test tubes with a volume of 5 mL. Add an equal volume of 1-phenyl-3-methyl-5-pyrazolone (PMP) methanol solution and mix thoroughly by vortexing. Place the tubes in a 70°C water bath for 2 h and then allow them to cool to room temperature.
5. Add 400 μL of 0.3 M HCl to adjust the pH to 6–7, followed by the addition of 1.2 mL of water. Add an equal volume of chloroform, mix well, and allow the mixture to settle. Discard the chloroform phase and repeat this process twice. Filter the aqueous phase through a 0.45 μm microporous membrane (aqueous phase) and proceed with HPLC injection analysis.

Mobile phase A consists of 100 mM sodium phosphate buffer (pH 6.7) as the aqueous phase, and mobile phase B consists of acetonitrile as an organic phase used in gradient mode. The initial gradient had 86% mobile phase A, and 14% mobile phase B intended for 9 min. Then, at 9 min the gradient was changed to consisting of 83% mobile phase A and 17% mobile phase B held for 19 min. From 28 min, a linear gradient was shifted to 78% mobile phase A as well as 22% mobile phase B, which was held at 1 min. At 29 min, the gradient reached 50% mobile phase A, and 50% mobile phase B held for 3 min. Then, the gradient was changed to consisting of 86% mobile phase A, and 14% mobile phase B at 32 min and held thus for 4 min to equilibrate before a fresh injection. The flow rate of the instrument was fixed at 1 mL per min. The analytical column used for assays was a 4.6 × 250 mm C18 column with a particle size of 5 μm and column temperature maintained at 30°C.The instrument was set at 5 μL for sample injection.

### 2.5 Cell culture and determination of cytotoxicity

HepG_2_ cells were thawed from-80°C freezer and cultured in DMEM complete medium containing 100 U/mL penicillin, 100 μg/mL streptomycin, and 10% fetal bovine serum (FBS). The cells were cultured in an incubator with 37°C in a 5% CO_2_ incubator. Accurately weigh 10 mg of the lyophilized *Armillaria gallica* polysaccharides and put them into a flask with a volume of 10 mL. 10 mL DMEM medium was added to make the 1 mg/mL stock concentration AgP. The stock AgP solution was filtered by 0.22 μm filter membrane. Then, the DMEM complete medium was used to distill the AgP to the treatment concentration (100, 200, 400 μg/mL).

The cells were divided into 5 groups, including the control group, H_2_O_2_ damage group and AgP pretreated group (100, 200, 400 μg/mL). Logarithmic phase HepG_2_ cells were collected and prepared as a single-cell suspension at a concentration of 1 × 10^5^ cells/mL, and were seeded into a 96-well plate with 100 μL per well. For a duration of 28 h, the HepG_2_ cells in the control group were cultured in a medium. AgP groups’ cells were cultivated in media with varying doses of AgP (100, 200, and 400 μg/mL). In the H_2_O_2_ damage group, HepG2 cells were cultured in a medium. The AgP group and the H_2_O_2_ damage group were treated with 0.9 mM H_2_O_2_ (dissolved in DMEM) or 4 h after being incubated for 24 h. Using an assay kit called the Cell Counting Kit-8 (CCK-8), cell viability was assessed. The cell viability was calculated using the following formula:


$$\mathrm{cell}\;\mathrm{viability}\;\left(\%\right)=\frac{\mathrm{the}\;\mathrm{absorbance}\;\mathrm{of}\;\mathrm{experimental}\;\mathrm{group}\;}{\mathrm{the}\;\mathrm{absorbance}\;\mathrm{of}\;\mathrm{natural}\;\mathrm{group}}\times 100\%$$

### 2.6 Detection of biochemical indexes of HepG_2_ cells

HepG_2_ cells were cultured in the conditions stipulated in section 2.5. The culture medium was collected and the levels of alanine aminotransferase (ALT) and lactate dehydrogenase (LDH) were measured following the instructions provided with the assay kit. The cells were collected by a cell scraper and transferred to plastic centrifuge tubes using a pipette. Cell suspension was prepared by sonicating the cells in an ice-water bath for 5 min at 300 W. The levels of malondialdehyde (MDA), superoxide dismutase (SOD), glutathione peroxidase (GSH-PX), catalase (CAT), as well as the activities of caspase-3, caspase-8, and caspase-9 were measured based on the instructions provided with the respective assay kits.

### 2.7 The detection of HepG_2_ cell apoptosis

HepG_2_ cells were cultured under conditions set in 2.5. After collecting the cells by digestion with trypsin, they were re-suspended in 1x Binding Buffer. The cells were then subjected to an apoptosis assay using the ANNEXIN V-FITC/PI Apoptosis Detection Kit, following the instructions provided with the kit. After incubation, the cells were detected by flow cytometer (Beckman Coulter China Co., Ltd.)

### 2.8 The detection of accumulated ROS in HepG_2_

HepG2 cells were cultured under conditions stipulated in 2.5. After collecting the cells by digestion with trypsin, HepG2 cells were re-suspended in 10 μmol/L DCFH-DA and the cell and DCFH-DA were kept in an incubator with 37°C in a 5% CO_2_ incubator for 20 min. The DCFH-DA was diluted by DMEM medium with the dilution ratio being 1:1000(DCFH-DA: DMEM). After the reaction, the cells were washed three times with DMEM and fluorescence was observed under an inverted fluorescence microscope (Shanghai Cewe Optoelectronic Technology Co., Ltd.).

### 2.9 Data processing

The data were processed using Excel and SPSS 21, with the mean ± standard deviation (X ± SD) as the descriptive statistics. Statistical analysis was performed using SPSS 21 and one-way ANOVA was adopted to assess the significance of the results, with a predetermined significance level of *p* < 0.05 to indicate statistical significance. The visual representations in this paper were generated using GraphPad 8.0.2.

## 3 Results and discussion

### 3.1 Monosaccharide composition of polysaccharides of *Armillaria gallica*

**Figure 1:**
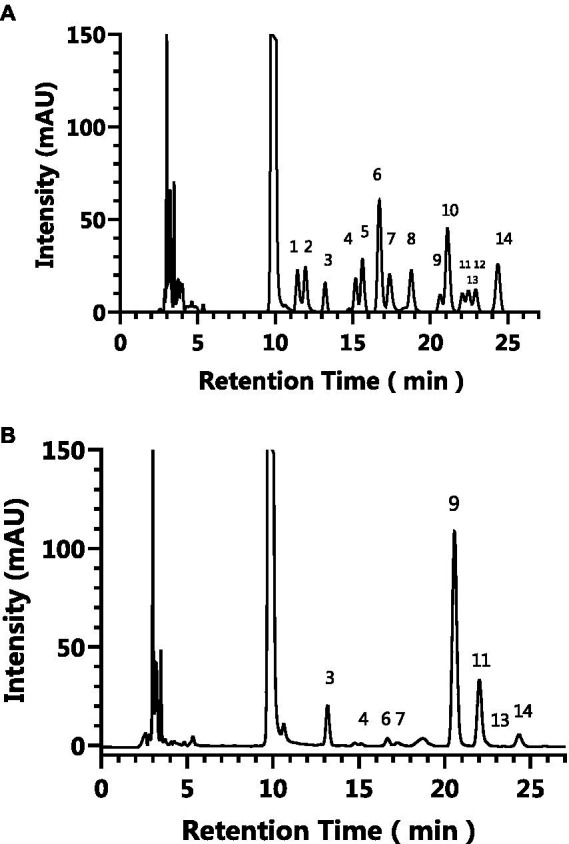
The HPLC results of the monosaccharide standards **(A)** and AgP **(B)**. The characteristic peaks are as follows: peak 1: guluronic acid, peak 2: mannuronic acid, peak 3: mannose, peak 4: ribose, peak 5: rhamnose, peak 6: glucosamine, peak 7: glucuronic aldehyde, peak 8: galacturonic aldehyde, peak 9: glucose, peak 10: aminogalactose, peak 11: galactose, peak 12: xylose, peak 13: arabinose, peak 14: L-fucose.

Pre-column PMP derivatization HPLC was used to analyze monosaccharide composition of AgP. Figure 1 depicts the liquid chromatography profiles of 14 monosaccharide standards and AgP. As shown in the figure, the elution order of the 14 monosaccharide standards is as follows: guluronic acid, mannuronic acid, mannose, ribose, rhamnose, glucosamine, glucuronic aldehyde, galacturonic aldehyde, glucose, aminogalactose, galactose, xylose, arabinose, L-fucose. By comparing the retention times and peak areas of each standard, the monosaccharides composition of the hydrolyzed polysaccharides of *Armillaria gallica* was determined and is presented in Table 1. The results indicate that AgP is composed of glucose, mannose, galactose, L-fucose, guluronic acid, glucosamine, ribose, and arabinose.

**Table 1:** Monosaccharide composition of AgP.

Monosaccharide mg/g	Mannose	Ribose	Glucosamine	Guluronic acid	Glucose	Galactose	Arabinose	L-fucose
	55.05 ± 9	3.05 ± 0.6	3.05 ± 1	4.90 ± 2	512.33 ± 40	151.00 ± 62	0.86	10.87 ± 4

### 3.2 Effects of H_2_O_2_ and AgP on HepG_2_ cells

**Figure 2:**
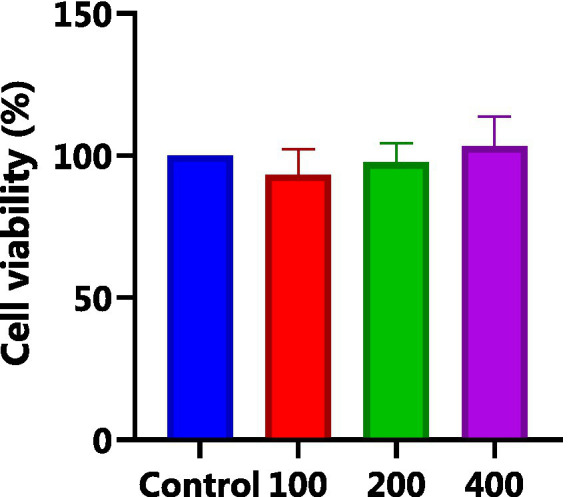
Effect of AGP on HepG_2_ cells. AgP pretreat HepG_2_ cells at the concentrations of 100, 200, 400 μg/mL for 24 h.

**Figure 3:**
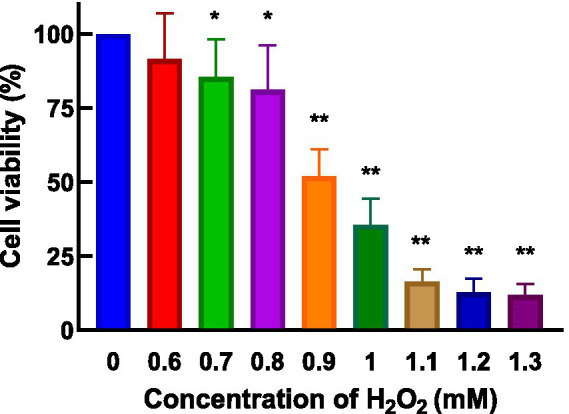
Effect of H_2_O_2_ on HepG_2_ cells. H_2_O_2_ damage HepG_2_ cells at different concentrations for 4 h.

HepG_2_ cells, isolated from human hepatocellular carcinoma, are highly differentiated, non-viral cells that exhibit similar metabolic and biotransformation activities as primary human liver cells (16). The impact of AgP on HepG_2_ cells is depicted in Figure 2. Within the concentration range of 100–400 μg/mL of AgP, the two polysaccharide components showed no significant effect on HepG_2_ cells, and cell viability remained above 90%. HepG_2_ cells can sustain oxidative damage from oxidizing agents, which can be reversed by using the right antioxidants (16). H_2_O_2_ easily penetrates the cell membrane and possesses strong oxidizing properties. H_2_O_2_ can react with cellular Fe^3+^ through the Fenton reaction and produce highly reactive oxygen species (ROS). Excessive ROS stimulation leads to oxidative stress in cells, where the generated ROS attacks the unsaturated fatty acids in the phospholipid membrane, triggering a cascade of lipid peroxidation reactions (17). Therefore, this study employed hydrogen peroxide to induce oxidative damage in the cell model. Figure 3 illustrates the effect of different concentrations of H_2_O_2_ on HepG_2_ cells. As shown in the figure, as the concentration of H_2_O_2_ increases, the cell viability of HepG_2_ cells gradually decreases. At 0.9 mM H_2_O_2_, the cell viability is 51.89%, approaching the IC50 value. Both excessively high and low cell viabilities can obscure the effects of polysaccharides on cells. Therefore, a concentration of 0.9 mM H_2_O_2_ was chosen to establish the H_2_O_2_-induced oxidative damage model in HepG_2_ cells.

### 3.3 Protective effect of AgP on H_2_O_2_-induced cell injury

**Figure 4:**
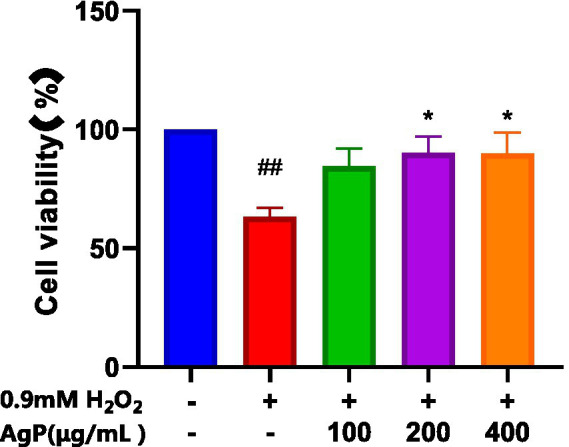
Protective effect of AgP on cell viability of H_2_O_2_-induced cell injury. The cells in control group were grown in the DMEM complete medium for 28 h. The cells in AgP groups (100, 200, 400 μg/mL) were pretreated by AgP for 24 h, and then were damaged by H_2_O_2_ for 4 h. The H_2_O_2_ group were added to DMEM complete medium, and then were decomposed by H_2_O_2_ for 4 h., respectively (+ represents addition; − represents without addition). Compared with the H_2_O_2_ group, ^*^p < 0.05, ^**^p < 0.01; compared with the control group, ^#^*p* < 0.05, ^##^*p* < 0.01.

As shown in Figure 4, AgP helps prevent H_2_O_2_-induced oxidative damage, which is concentration-dependent. It has been found that mannose may play an important role in the antioxidant activity of polysaccharides. The aberrant leakage of ALT and LDH in HepG_2_ cells increased significantly (*p* 0.05) following H_2_O_2_ treatment, demonstrating the viability of the H_2_O_2_-induced oxidative damage model (18). After H_2_O_2_ treatment, abnormal leakage of ALT and LDH in HepG_2_ cells significantly increased (*p* < 0.05), indicating the successful establishment of the H_2_O_2_-induced oxidative damage model. Under the influence of AgP, the abnormal leakage of ALT and LDH in HepG_2_ cells significantly decreased (*p* < 0.05). This may be attributed to the fact that H_2_O_2_ damages HepG_2_ cell membranes, disrupts the cell membrane (19, 20), increases cell permeability, and causes ALT and LDH to be released into the extracellular space. AgP treatment of HepG_2_ cells can reduce the abnormal leakage of ALT and LDH caused by H_2_O_2_, possibly due to the mannose content in AgP, which can form a protective membrane (18).

### 3.4 ROS accumulation in HepG_2_ cells

**Figure 5:**
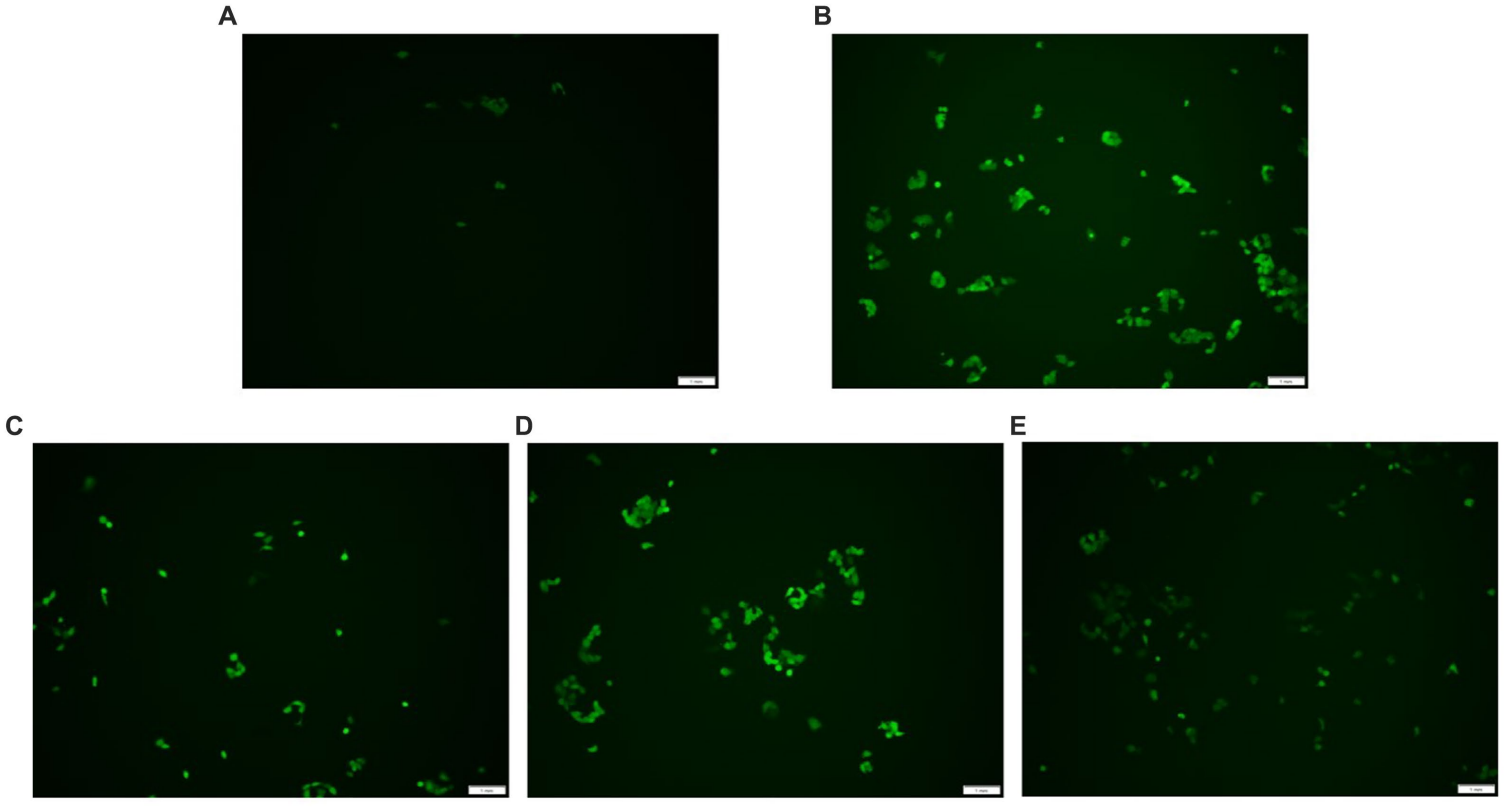
ROS detection results. **(A)** control group; **(B)** H_2_O_2_ group; **(C)** AgP 100 μg/mL group; **(D)** AgP 200 μg/mL group; **(E)** AgP 400 μg/mL group. The cells in control group were grown in the DMEM complete medium for 28 h. The cells in AgP groups were pretreated by AgP for 24 h, and then were decomposed by H_2_O_2_ for 4 h. The H_2_O_2_ group were added to DMEM complete medium, and then were decomposed by H_2_O_2_ for 4 h. The scale-bar is 1 mm.

DCFH-DA can freely penetrate the cell membrane and does not glow itself. Esterases hydrolyze DCFH-DA once they are within the cell to produce DCFH. The cell membrane prevents DCFH from passing through it, making it simple to load the probe inside the cell. DCFH is oxidized by intracellular ROS, producing DCF, which is a fluorescent molecule. The quantity of reactive oxygen species (ROS) in the cell is shown by the fluorescence intensity of DCF. Higher fluorescence intensity shows higher ROS levels. As indicated in Figure 5, the fluorescence intensity of the H_2_O_2_ group was stronger than the control group, but the AgP pretreated groups exhibited weaker fluorescence intensity compared with the H_2_O_2_ damage group. Adding AgP can improve the elevated ROS levels caused by H_2_O_2_ and exhibits a concentration-dependent effect. These results further confirmed that AgP was endowed with ROS scavenging ability and excellent antioxidant activity *in vitro*.

### 3.5 Determination results of Alt and LDH in HepG_2_ cells

**Figure 6:**
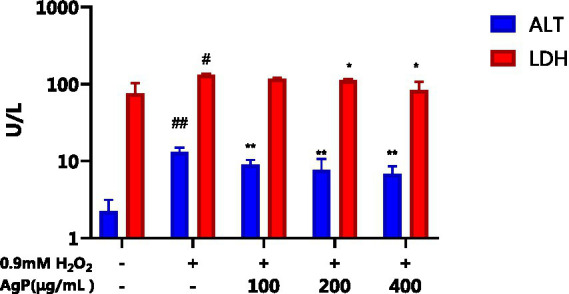
Effect of AgP on ALT and LDH. The cells in control group were grown in the DMEM complete medium for 28 h. The cells in AgP groups (100, 200, 400 μg/mL) were pretreated by AgP for 24 h, and then were damaged by H_2_O_2_ for 4 h. The H_2_O_2_ group were added to DMEM complete medium, and then were damaged by H_2_O_2_ for 4 h (+ represents addition; − represents without addition). In the H_2_O_2_ group, ^*^p < 0.05, ^**^p < 0.01; The control group, ^#^*p* < 0.05, ^##^*p* < 0.01.

The H_2_O_2_-induced oxidative damage model has been successfully established, as can be shown in Figure 6, where there is a significant increase in the aberrant leakage of ALT and LDH in HepG_2_ cells following H_2_O_2_ treatment (*p* < 0.05). Under the influence of AgP, the abnormal leakage of ALT and LDH in HepG_2_ cells significantly decreased (*p* < 0.05). Intracellular LDH and ALT leaked from intracellular to extracellular due to cytotoxicity by H_2_O_2_. H_2_O_2_ treatment greatly improved LDH and ALT levels, and the AgP treatment can prevent the cytotoxicity from H_2_O_2_. This might be attributed to the fact that H_2_O_2_ damages HepG_2_ cell membranes (19, 20), increasing cell permeability, and causing ALT and LDH to be released into the extracellular space. AgP treatment of HepG_2_ cells can reduce the abnormal leakage of ALT and LDH caused by H_2_O_2_, which can be correlated to the mannose content in AgP that can form a protective membrane (18).

### 3.6 Effects of AgP pretreatment in H2O2-induced HepG2 cells on MDA content, sod, cat, and GSH-Px activities

The elements most closely linked to the antioxidant properties of HepG_2_ cells include ROS, MDA, and antioxidant enzymes (SOD, GSH-PX, CAT) (16). SOD serves as the primary defense against oxygen free radicals, protecting cells from oxidative damage and lipid peroxidation by scavenging free radicals (17). GSH-Px is a peroxide decomposing enzyme and can catalyze the reduction of peroxides, such as H_2_O_2_, into non-toxic hydroxyl compounds by GSH (21). CAT, as an essential enzyme system responsible for removing H_2_O_2_ within the body, interacts with intracellular mitochondria and peroxidases. Together with GSH-PX, it quickly removes harmful byproducts of cellular metabolism including hydrogen peroxide while promoting detoxification procedures and safeguarding membrane proteins and thiol enzymes. ROS have the ability to attack polyunsaturated fatty acids (PUFAs) in biological membranes, starting the process of lipid peroxidation and producing malondialdehyde (MDA). The ability of cells to withstand oxidation may be reflected in changes in the activity of several internal antioxidant enzymes. Higher MDA levels indicate more severe oxidative damage to the cells, which can be used as an indirect indicator of the extent of cellular damage produced by ROS (14, 21).

**Figure 7:**
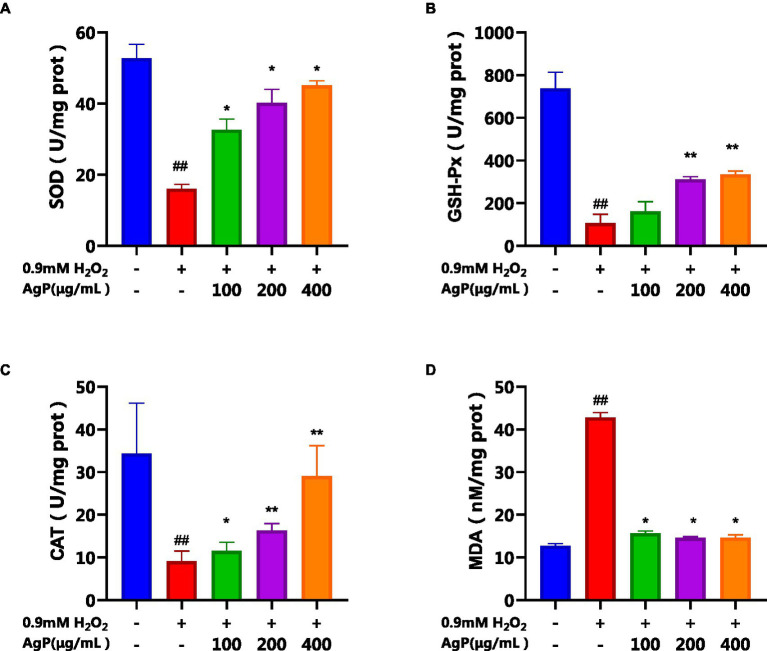
Effects of AgP on the levels of antioxidant enzyme (**A**: SOD, **B**: GSH-Px, **C**: CAT) and **(D)** MDA in HepG2 cells (+ represents addition; − represents without addition). The cells in control group were grown in the DMEM complete medium for 28 h. The cells in AgP groups (100, 200, 400 μg/mL) were pretreated by AgP for 24 h, and then were damaged by H_2_O_2_ for 4 h. The H_2_O_2_ group was added to DMEM complete medium, and then was damaged by H_2_O_2_ for 4 h. In the H_2_O_2_ group, ^*^p < 0.05, ^**^p < 0.01; In the control group, ^#^*p* < 0.05, ^##^*p* < 0.01.

As observed in Figure 7, under the influence of H_2_O_2_, the activity of antioxidant enzymes (SOD, GSH-PX, CAT) in HepG_2_ cells significantly decreased, while the MDA content significantly increased (*p* < 0.05). However, upon the addition of different concentrations of AgP, the activities of SOD, GSH-PX, and CAT were significantly higher than those in the model group, and the MDA content was significantly lower (*p* < 0.05). Further evidence that AgP can control the activity of antioxidant enzymes to ward off oxidative damage brought on by H_2_O_2_ came from the fact that this impact showed a concentration-dependent relationship (22). The free radicals were produced and accumulated in H_2_O_2_-treated HepG2 cells, brought about lipid and protein peroxidation of cellular membranes and led to cell death (21). However, the intracellular antioxidant enzyme can catalyze the disproportionation reaction of superoxide ion radicals to generate H_2_O_2_ and water, thereby scavenging superoxide ion radicals and alleviating the oxidative damage in HepG2 cells (21). The results showed that AgP might reduce oxidative damage to cells by scavenging reactive oxygen species (ROS), activated intracellular antioxidant enzymes, and prevented lipid molecules from oxidizing.

### 3.7 Detection of HepG_2_ cell apoptosis results using flow cytometry

**Figure 8:**
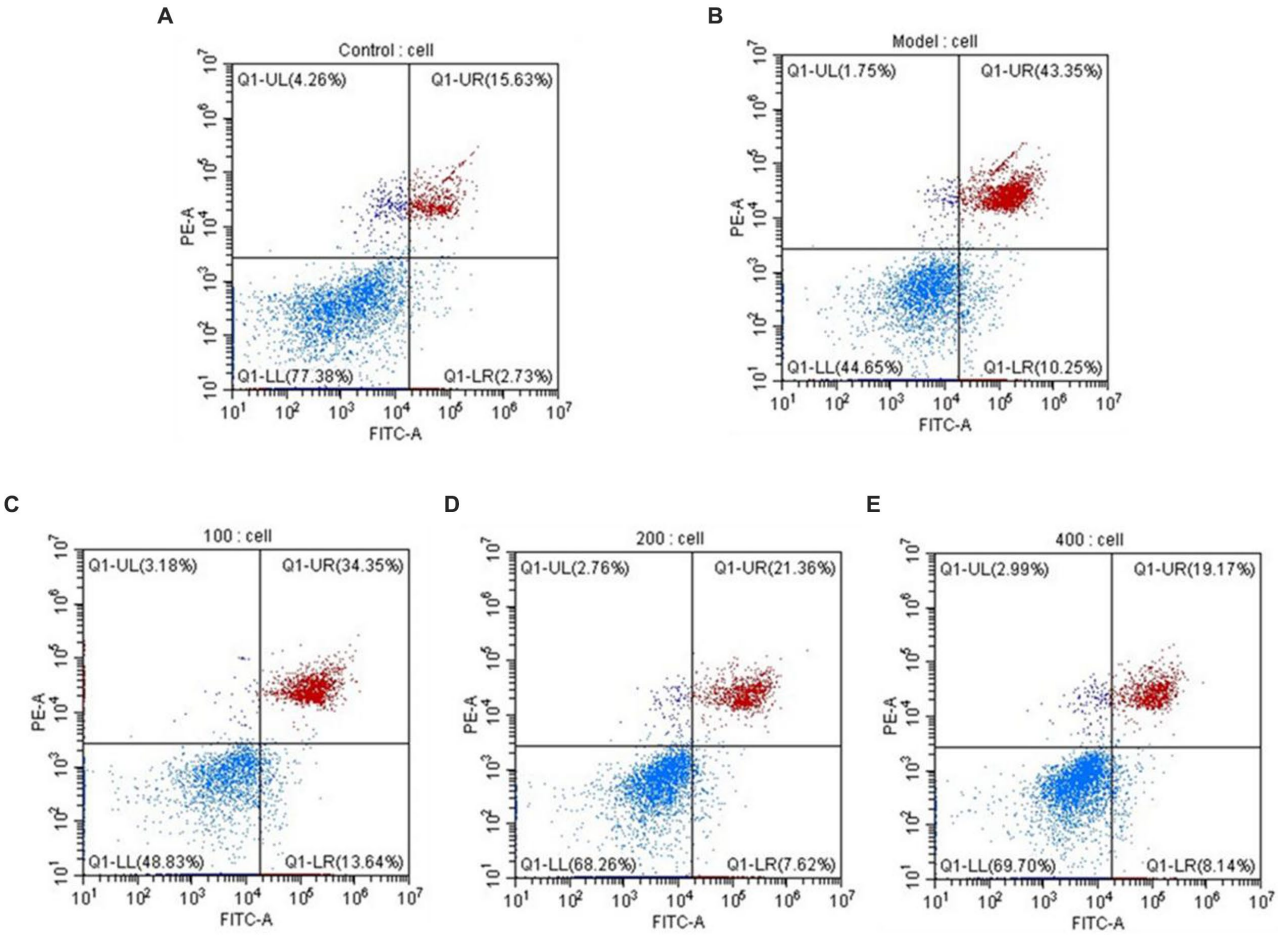
The apoptosis rate of HepG_2_ cells. **(A)** control group; **(B)** H_2_O_2_ group; **(C)** AgP 100 μg/mL group; **(D)** AgP 200 μg/mL group; **(E)** AgP 400 μg/mL group. The cells in control group were grown in the DMEM complete medium for 28 h. The cells in AgP groups were pretreated by AgP for 24 h, and then were damaged by H_2_O_2_ for 4 h. The H_2_O_2_ group was added to DMEM complete medium, and then were damaged by H_2_O_2_ for 4 h.

During cellular apoptosis, phosphatidylserine (PS) flips to the outer membrane. Annexin V, a Ca^2+^-dependent phospholipid-binding protein, exhibits high affinity for PS and can specifically bind to it. When FITC-Annexin V is attached to apoptotic cells, it exhibits green fluorescence when excited by blue light, allowing apoptotic cells to be distinguished from healthy cells. The nucleus of late-stage apoptotic cells and dead cells can be stained red by the nucleic acid stain propidium iodide (PI), although undamaged cell membranes are impermeable to it. Early-stage and late-stage apoptotic cells can be distinguished using FITC-Annexin V and PI. The results, as shown in Figure 8, indicate that under the influence of H_2_O_2_, the percentage of late-stage apoptotic cells is 43.35%, while the percentage of early-stage apoptotic cells is 10.25%, supporting the successful construction of an H_2_O_2_-induced oxidative damage model in HepG_2_ cells. AgP has a concentration-dependent effect on the reduction of late-stage apoptotic HepG_2_ cell percentage. AgP can prevent oxidative damage-induced apoptosis in HepG_2_ cells brought on by H_2_O_2_ by reducing the percentage of late-stage apoptosis, which is 19.17% at a dosage of 400 μg/mL.

### 3.8 Determination of caspase-3, 8, 9 activity in HepG_2_ cells

**Figure 9:**
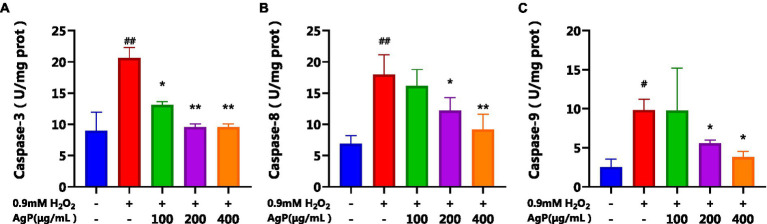
Effect of AgP on Caspase-3**(A)**, Caspase-8 **(B)** and Caspase-9 **(C)** activity in HepG_2_ cells (+ represents addition; − represents without addition). The cells in control group were grown in the DMEM complete medium for 28 h. The cells in AgP groups (100, 200, 400 μg/mL) were pretreated by AgP for 24 h, and then were damaged by H_2_O_2_ for 4 h. The H_2_O_2_ group were added to DMEM complete medium, and then were damaged by H_2_O_2_ for 4 h. In the H_2_O_2_ group, ^*^p < 0.05, ^**^p < 0.01;in the control group, ^#^*p* < 0.05, ^##^*p* < 0.01.

By regulating and managing cellular activities, caspases assist in maintaining cellular homeostasis, which is essential for preserving a healthy physiological metabolism and tissue homeostasis. (23). Both intrinsic and extrinsic apoptotic processes involve caspases as important players (23). During apoptosis activation, Caspase-8 serves as an upstream caspase in the process of cell apoptosis. Caspase-8 is activated and forms dimers in the apoptotic processes mediated by the Fas-receptor and TNFR-1, which in turn activates downstream caspases, including caspase. (22). Cytochrome c (CytC) and Apsf1 can combine to produce a complex that activates caspase-9, also known as ICE-LAP6 or Mch6. Caspase-3 is the most significant terminal protease in the process of apoptosis. Through specific hydrolysis, it activates procaspases 2, 6, 7, and 9, which causes the cleavage of several important apoptotic proteins like PARP. According to studies, the mechanism underlying the induction of cellular apoptosis in HepG_2_ cells by oxidative damage involves a reduction in the activity of antioxidant enzymes, leads to oxidative injury and increases the activity of caspase-9 and caspase-3 proteases (20, 24, 25). Analysis of Figure 9 reveals that under the influence of H_2_O_2_, there is a significant augmentation (*p* < 0.05) in the activity of caspase-8, 9, and 3, which can induce cellular apoptosis. However, AgP at concentrations of 200 μg/mL and 400 μg/mL effectively mitigates (*p* < 0.05) the H_2_O_2_-induced elevation in caspase-8 and caspase-9 activity. Similarly, AgP at concentrations of 100 μg/mL, 200 μg/mL, and 400 μg/mL significantly attenuates (p < 0.05) the H_2_O_2_-induced increase in caspase-3 activity, showing a concentration-dependent effect that aligns with the findings of flow cytometry analysis. These results strongly indicate that AgP enhances the activity of antioxidant enzymes and mitigates ROS-mediated cellular apoptosis, thereby exerting a protective effect.

## 4 Conclusion

At the cellular level, an oxidative damage model of HepG_2_ cells was established using a concentration of 0.9 mM H_2_O_2_. The protective effect of AgP on the H_2_O_2_-induced oxidative damage model in HepG_2_ cells was investigated, and it was found that AgP exhibits a protective effect on HepG_2_ cells.

The protective properties of AgP against H_2_O_2−_induced oxidative damage in HepG_2_ cells were further investigated, and it was discovered that AgP, at concentrations between 200 and 400 μg/mL, is capable of lowering ROS expression in HepG_2_ cells. Moreover, AgP significantly decreases the abnormal leakage of LDH and ALT induced by H_2_O_2_, thereby protecting the cell membrane. Additionally, AgP regulates the activity of antioxidant enzymes, such as SOD, CAT, and GSH-PX, and reduces the level of MDA to protect cells from damage. Flow cytometry analysis and measurement of caspase-3, 8, and 9 activities reveal that AgP can modulate apoptosis-related proteins and attenuate ROS-induced cellular apoptosis. In summary, AgP enhances the activity of antioxidant enzymes and mitigates ROS-induced cellular apoptosis, thereby exerting a protective effect.

## Data availability statement

The original contributions presented in the study are included in the article/supplementary materials, further inquiries can be directed to the corresponding author.

## Author contributions

PS: Conceptualization, Investigation, Writing – original draft. HQ: Data curation, Validation, Writing – original draft. LL: Formal analysis, Software, Writing – review & editing. LUW: Methodology, Writing – review & editing. YL: Supervision, Writing – review & editing. JL: Resources, Methodology, Writing – review & editing. LIW: Visualization, Methodology, Writing – review & editing. FM: Investigation, Methodology, Writing – review & editing.
